# Inflammatory Breast Cancer: Clinical Implications of Genomic Alterations and Mutational Profiling

**DOI:** 10.3390/cancers12102816

**Published:** 2020-09-30

**Authors:** Flávia L. C. Faldoni, Rolando A. R. Villacis, Luisa M. Canto, Carlos E. Fonseca-Alves, Sarah S. Cury, Simon J. Larsen, Mads M. Aagaard, Cristiano P. Souza, Cristovam Scapulatempo-Neto, Cynthia A. B. T. Osório, Jan Baumbach, Fabio A. Marchi, Silvia R. Rogatto

**Affiliations:** 1 International Research Center, A.C.Camargo Cancer Center, São Paulo 01508-010, Brazil; flaviafaldoni@gmail.com (F.L.C.F.); fabio.marchi@accamargo.org.br (F.A.M.); 2 Department of Clinical Genetics, University Hospital of Southern Denmark, 7100 Vejle, Denmark; luisa.matos.do.canto.alvim@rsyd.dk (L.M.C.); mads.jorgensen@rsyd.dk (M.M.A.); 3 Department of Genetics and Morphology, Institute of Biological Sciences, University of Brasília-UnB, Brasília 70910-900, Brazil; rolando.andre@unb.br; 4 Department of Veterinary Surgery and Anesthesiology, School of Veterinary Medicine and Animal Science, São Paulo State University-UNESP, Botucatu 18618-681, Brazil; carlos.e.alves@unesp.br; 5 Department of Structural and Functional Biology, Institute of Biosciences, São Paulo State University-UNESP, Botucatu 18618-689, Brazil; santiloni.cury@unesp.br; 6 Department of Mathematics and Computer Science, University of Southern Denmark, 5230 Odense, Denmark; sjlarsen@imada.sdu.dk (S.J.L.); jan.baumbach@wzw.tum.de (J.B.); 7 Department of Breast and Gynecologic Oncology, Barretos Cancer Hospital, Pio XII Foundation, Barretos 14784-390, Brazil; crispeixoto10@hcancerbarretos.com.br; 8 Molecular Oncology Research Center, Barretos SP 14784-400, Brazil; cristovam.neto.ext@dasa.com.br; 9 Diagnósticos da América (DASA), Barueri 01525-001, Brazil; 10 Department of Pathology, A.C.Camargo Cancer Center, São Paulo 01525-001, Brazil; cabtoledo@accamargo.org.br; 11 TUM School of Life Sciences Weihenstephan, Technical University of Munich, 85354 Freising, Germany; 12 Institute of Regional Health Research, University of Southern Denmark, 500 Odense, Denmark

**Keywords:** inflammatory breast cancer, microarray, gene variants, genomic scars, homologous recombination deficiency, copy number alterations

## Abstract

**Simple Summary:**

Inflammatory breast cancer (IBC) is an aggressive disease with high mortality rates. Nowadays, there is no targeted treatment for this tumor type. Based on this context, we investigated the molecular profile of this disease by using well-established methodologies (high-resolution microarray platform, targeted next-generation sequencing, and immunohistochemistry) that have proven potential to unveil cancer biomarkers. We found alterations related to IBC aggressiveness and metastasis (gains of *MDM4*, losses of *CHL1*, and high homologous recombination deficiency scores), and worse overall survival (variants in HR and mismatch repair genes). We also compared the mutational profiling of our cases with literature data, which includes both non-IBC and IBC cases, validating our findings. Overall, we describe genetic alterations with the potential to be used as prognostic or predictive biomarkers and ultimately improve IBC patients’ care.

**Abstract:**

Inflammatory breast cancer (IBC) is a rare and aggressive type of breast cancer whose molecular basis is poorly understood. We performed a comprehensive molecular analysis of 24 IBC biopsies naïve of treatment, using a high-resolution microarray platform and targeted next-generation sequencing (105 cancer-related genes). The genes more frequently affected by gains were *MYC* (75%) and *MDM4* (71%), while frequent losses encompassed *TP53* (71%) and *RB1* (58%). Increased MYC and MDM4 protein expression levels were detected in 18 cases. These genes have been related to IBC aggressiveness, and MDM4 is a potential therapeutic target in IBC. Functional enrichment analysis revealed genes associated with inflammatory regulation and immune response. High homologous recombination (HR) deficiency scores were detected in triple-negative and metastatic IBC cases. A high telomeric allelic imbalance score was found in patients having worse overall survival (OS). The mutational profiling was compared with non-IBC (TCGA, *n* = 250) and IBC (*n* = 118) from four datasets, validating our findings. Higher frequency of *TP53* and *BRCA2* variants were detected compared to non-IBC, while *PIKC3A* showed similar frequency. Variants in mismatch repair and HR genes were associated with worse OS. Our study provided a framework for improved diagnosis and therapeutic alternatives for this aggressive tumor type.

## 1. Introduction

Inflammatory breast cancer (IBC) stands out as a rare and aggressive form of breast cancer (BC), accounting for only 2–4% of BC patients, but responsible for 7–10% of BC-related deaths [1,2,3]. The IBC diagnosis is based on inflammatory clinical signs (warmth, erythema, edema-peau d’orange, lasting no more than 6 months, with erythema occupying at least one-third of the breast) in more than 30% of the breast, with or without an underlying palpable mass [2,3,4,5]. Dermal lymphatic invasion is a non-obligatory histopathological feature supporting the diagnosis of IBC [2,3,4,5]. The lymph vascular tumor emboli, identified in ~75% of IBC patients, contributes with aggressive behavior, strong metastatic potential, and poor prognosis [2,4,6]. Approximately 30% of newly diagnosed cases have distant metastases [4]. The median overall survival (OS) of patients with IBC stages III and IV is shorter compared to non-IBC cases (stage III: 4.75 versus 13.40 years and stage IV: 2.27 versus 3.40 years, respectively) [7,8].

The rarity of the disease, misdiagnosis, and difficulty in sample collection before treatment have resulted in a limited number of published molecular studies [2,5,9,10]. Transcriptome profiling analyses revealed that IBC is highly heterogeneous, showing the same molecular subtypes of non-IBC, with a higher proportion of HER2-positive and triple-negative (TNBC: negativity for estrogen receptor: ER, progesterone receptor: PR, and HER2) subtypes [2,5,11,12,13].

Copy number alterations (CNAs) have been reported in a few studies using both low-resolution techniques (such as restriction fragment length polymorphism or microsatellite markers) and more robust methodologies (array-comparative genomic hybridization and next-generation sequencing (NGS) [10,14,15,16,17,18]. CNA data also failed in distinguishing IBC from non-IBC cases. Nevertheless, some chromosomal regions were more frequently altered in IBC, including gains of 1q, 8q, and 17q [16]. Compared to non-IBC, IBC displayed a more complex pattern of chromosomal imbalances indicating a higher genomic instability [16]. The mutational profile of IBC assessed by NGS revealed higher mutation rates of *TP53*, *PIK3CA, NOTCH1*, and *BRCA2,* among other alterations [9,10,18,19]. The identification of clinically relevant somatic variants may be useful for developing novel and effective targeted therapies, which may improve the survival rates of IBC patients [9,10,18,19].

In the last decade, several microarray-based signatures have been developed to measure chromosomal instability (CIN), a hallmark of most solid tumors [20,21,22]. CIN is characterized by increased levels of CNAs covering large fragments of chromosomes [22]. The genomic instability index (GII) estimates the genome fraction affected by CNAs and might be associated with homologous recombination deficiency (HRD) in sporadic BC [22]. Genomic scar signatures associated with HRD; namely TAI (telomeric allelic imbalance), LST (large scale transition), and HRD-LOH (loss of heterozygosity), have been described as useful tools to identify BRCA1/2-deficient BC, which in turn could benefit the patients with platinum or PARP (Poly ADP-Ribose Polymerase) inhibitor therapies [10,20,21,23].

The biological basis of IBC still remains poorly understood [5]. In this context, we molecularly explored a cohort of 24 IBC samples using a high-resolution microarray platform (2.6 million probes) and targeted-NGS (tNGS) of 105 cancer-related genes. We described potential prognostic and predictive biomarkers that may contribute to a better understanding of IBC development and progression.

## 2. Results

### 2.1. Clinical and Pathological Characteristics of IBC Patients

The clinicopathological information of 34 women diagnosed with IBC was retrieved from medical records (Table 1). Ten cases were used exclusively in the IHC experiments. Most patients (*n* = 24) aged 50 years or older at diagnosis (median age of 51, ranging from 36–82 years-old) presented a family history of cancer (*n* = 23). According to the body mass index (BMI), 25 patients were considered overweight or obese (BMI ≥ 25). Twenty-one patients had clinical stage III at diagnosis, and 19 IBC samples presented histological grade III. Distant metastases were identified in 26 patients, 13 at diagnosis (clinical stage IV), and 13 during follow-up. Twenty-five women died at a medium time of 25.3 months (3.1 to 105.2 months). Eighteen IBC cases were hormone receptors (estrogen and/or progesterone receptors) positive and HER2 negative, 11 were triple negatives, and 5 patients had hormone receptors negative and HER2 positive. IBC cases classified as hormone receptors and HER2 positives were not detected in our sample set.

Patients with clinical stage III (*n* = 19) were initially treated with neoadjuvant chemotherapy with four cycles of doxorubicin and cyclophosphamide, followed by paclitaxel for 12 weeks. For HER2+ IBC patients, trastuzumab was also administered. Additionally, some hormone receptor-positive IBC patients also received tamoxifen or anastrozole. Patients that were alive after the neoadjuvant treatment were referred to surgery and adjuvant radiotherapy. Only one patient (IBC26) presented a pathological complete response (pCR) (absence of cancer cells in all specimens evaluated) after the neoadjuvant treatment. IBC patients at Stage IV were referred to palliative chemotherapy with cycles of fluorouracil, doxorubicin, and cyclophosphamide. From this cohort of 34 patients, 24 and 21 cases were evaluated by microarray and tNGS experiments, respectively. Protein expression analysis for selected genes was evaluated in 18 cases, eight of them were also evaluated by microarray and seven by tNGS.

### 2.2. Genomic Profile-CNAs, cnLOH, and Chromothripsis (CTH)

We detected an average of ~82 CNAs per case (1267 gains, 704 losses, and 165 cnLOH). Eighteen cases presented more than 50 CNAs (Table S1). At least one cnLOH was identified in 22 IBC cases with frequent involvement of 3p, 6q, and Xq (Table S2). Four cases (IBC11, IBC15, IBC16, and IBC22) had more than 10 cnLOH, each affecting different chromosomal regions (Table S1).

**Figure 1:**
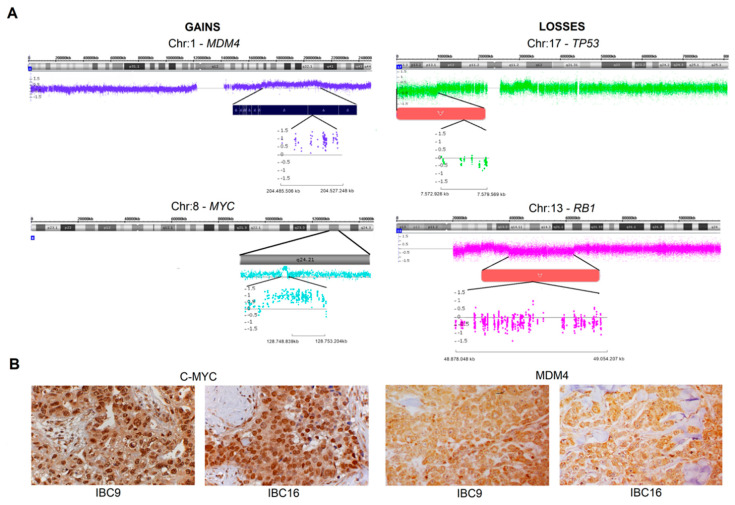
(**A**) Chromosomal location of some of the most common genes covered by gains (MYC and MDM4) and losses (RB1 and TP53) in inflammatory breast carcinomas. (**B**) Representative examples of high expression of C-MYC and MDM4 proteins in two cases (IBC9 and IBC16) that also showed MYC and MDM4 gains in the microarray analysis. It is possible to observe diffuse C-MYC and MDM4 expression in both cases. Hematoxylin counterstaining, 400×.

The most common gains were mapped in five chromosomes (1q, 7q, 8q, 17q, and 20q), whereas the losses were detected in seven chromosomes (3p, 8p, 9p, 13q, 17p, 19p, and 22q) (Table S2). The genes more frequently affected by copy number gains were *MYC* (18 cases) and *MDM4* (17 cases), and frequent losses encompassed *TP53* (17 cases) and *RB1* (14 cases) (Figure 1A). In triple-negative tumors, 85.7% (6/7) had *MDM4* and *MYC* gains. An in silico enrichment analysis using the set of genes frequently involved in gains and losses showed three of the 10 top enriched non-redundant biological processes related to inflammatory regulation, including humoral immune response, acute inflammatory response, and organ or tissue-specific immune response (*p* < 0.05) (Table S3).

The CNA profile was compared among patients stratified by the hormone receptors/HER2 status and presence of metastasis. Fifteen gains in six different chromosomes (2p, 5p, 6p, 11q, 17q, and 18) were more frequently detected (*p* < 0.01) in TNBC than in hormone receptor-positive and/or HER2+ tumor subtypes (Table S4). Furthermore, gains mapped on 8q (8q21and 8q24.11-q24.13) were found more frequently in metastatic patients compared to those without metastases (Table S5). Additional comparisons between clinical features and genomic alterations were not statistically significant.

Fourteen CTH events were detected in nine cases affecting chromosomes 8 (IBC7 and IBC14), 11 (IBC4, IBC7, and IBC24), 13 (IBC9 and IBC18), and 17 (IBC8 and IBC18) (Table S6). The presence of CTH was not statistically different for any of the clinicopathological parameters evaluated.

### 2.3. MDM4 and C-MYC Proteins Expression

A large number of cases showing *MDM4* (mapped in 1q23.1, 17 cases) and *MYC* (mapped in 8q24.21, 18 cases) gains were detected in our genomic analysis. Based on these findings, we investigated MDM4 and C-MYC proteins expression by immunohistochemistry in 18 available formalin-fixed paraffin-embedded IBC samples. All cases showed high expression levels of C-MYC and MDM4 (Figure 1B). C-MYC presented low to moderate cytoplasmic expression and moderate to high nuclear expression, while MDM4 had moderate to high cytoplasmic and nuclear expression. Eight of 18 samples were also evaluated by CNAs, and a set of them presented gains covering *MDM4* (six cases) and *MYC* (seven cases) genes, respectively. The Spearman test only found a positive correlation between *MYC* copy number state and C-MYC nuclear distribution scores (*r* = 0.8452, *p* = 0.0357).

### 2.4. Genomic Instability (GII) Analysis and HRD-Related Genomic Scars Signatures

**Figure 2:**
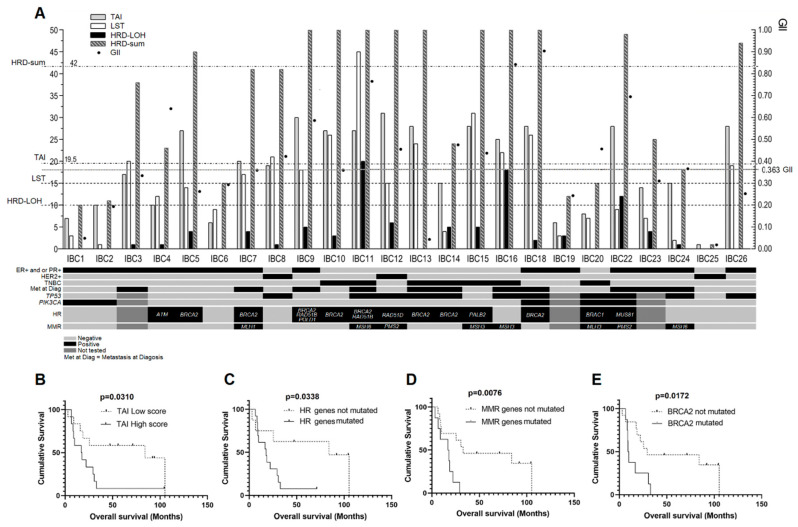
Graphical representation of homologous recombination deficiency (HRD)-associated scores (TAI, LST, HRD-LOH, and HRD-sum) and GII. (**A**) The dashed lines represent the thresholds for high and low scores. Cases with variants in homologous recombination (HR) genes, TP53, PIK3CA and mismatch repair (MMR) genes are also indicated. For the HR and MMR genes, the specific altered genes are shown, while variants in *TP53* and *PIK3CA* are represented by the plus sign (+). For technical issues, IBC3, IBC9, and IBC23 were not submitted to sequencing analysis. (**B**) Significant differences in overall survival curves according to TAI status. (**C**) HR variants. (**D**) MMR variants. (**E**) *BRCA2* variants.

Based on the median GII value (0.363), 12 cases presented high chromosomal instability (Figure 2A and Table S7). A tendency toward significance was found in cases with high genomic instability and the occurrence of CTH (*p* = 0.0894).

The median values for the TAI, LST, HRD-LOH and HRD-sum scores were 19.5, 14.5, 2.5, and 41, respectively. High score values were detected in 12 cases for TAI, 11 for LST and 11 for HRD-sum scores. Eight cases presented both high values of LST and HRD-SUM scores. High HRD-LOH scores were detected only in three cases (Figure 2A and Table S7). High positive correlation was observed between TAI and LST (*r* = 0.7024; *p* < 0.001); TAI and HRD-sum (*r* = 0.8536; *p* < 0.001); and LST and HRD-sum scores (*r* = 0.8994; *p* < 0.001) (Table S8).

Clinicopathological features showed no differences according to high HRD scores. However, the median values of TAI, LST, and HRD-sum scores were higher in TNBC patients (TAI: 27, LST: 24 and HRD-sum: 56) compared to hormone receptor-positive/HER2 negative and hormone receptor-negative/HER2 positive IBC cases (TAI: 17, LST: 12 and HRD-sum: 38) (Figure 2A). Similarly, higher scores were more frequent in metastatic cases (TAI: 25, LST: 15, and HRD-sum: 45) compared to those without metastases (TAI: 10, LST: 9 and HRD-sum: 15). A statistically significantly worse OS was only found in patients with high TAI compared to those with low TAI scores (*p* = 0.0310) (Figure 2B).

### 2.5. Mutational Profile

**Figure 3:**
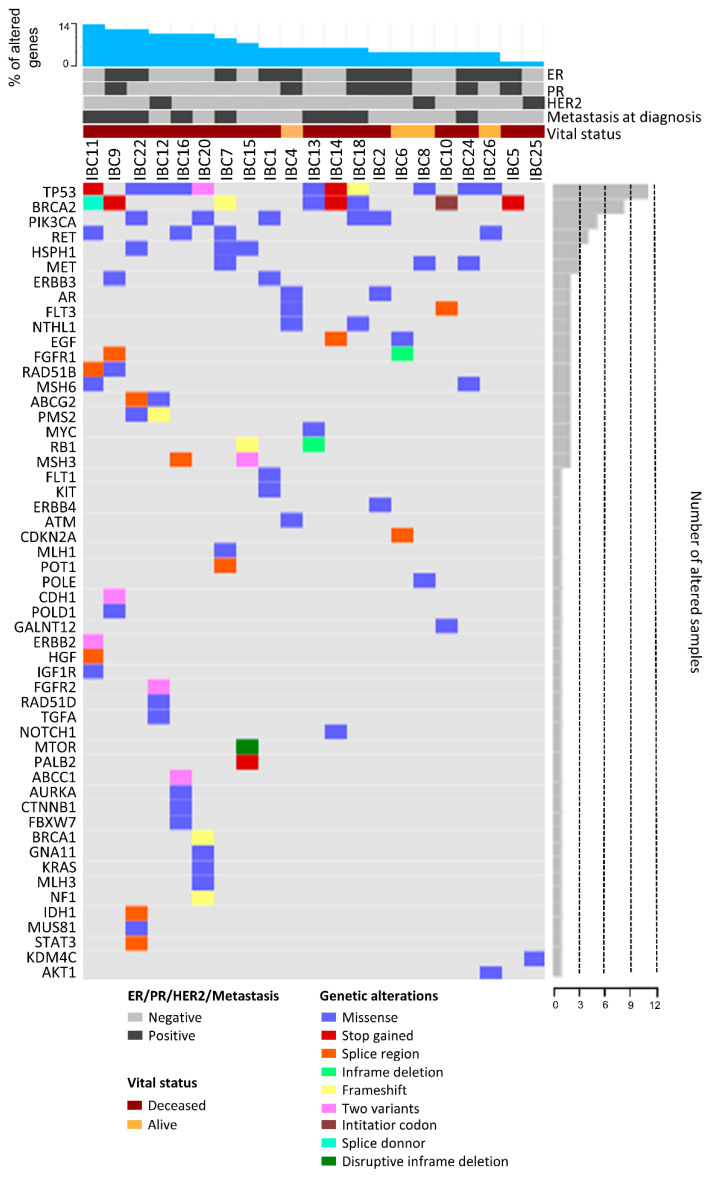
OncoPrint showing the 53 genes affected by mutations in the 21 IBC patients evaluated by targeted next-generation sequencing (tNGS). Patients from left to right present a decreased number of mutations (top panel), while genes from top to bottom were found mutated in a decreasing number of patients (right panel).

We detected 99 different variants in 53 of 105 cancer-related genes (Table S9) assessed by tNGS (Figure 3, Figure S1A and Table S10). All cases presented variants in at least one of the genes tested. The most common variants were found in *TP53* (11 cases), *BRCA2* (eight cases), and *PIK3CA* (five cases) (Table 2). The *PIK3CA* c.3140A > G variant was found in two IBC samples. Thirteen IBC samples presented variants in at least one gene of the HR pathway, especially in *BRCA2* (eight cases) (Figure 2A and Table 2). Similarly, eight IBC presented MMR variants (Table 2).

Metastatic IBC cases were enriched with variants in HR genes (*p* = 0.05) (Figure 2A). OS was significantly worse in patients having HR (*p* = 0.0338) (Figure 2C) or MMR variants (*p* = 0.0076) (Figure 2D). A multivariate analysis was performed considering OS, presence of metastasis, hormone receptors/HER2 status, and variants in HR genes. This analysis demonstrated an association of OS with metastasis (*r* = −0.47, *p* = 0.05) and HR genes variants (*r* = −0.42, *p* = 0.02) as an independent manner. Furthermore, worse OS was found in cases with *BRCA2* variants compared to those with BRCA2 wild type (*p* = 0.0172) (Figure 2E). No significant association was detected comparing CTH and MMR (*p* > 0.9999), HR (*p* = 0.3999), *TP53* (*p* > 0.9999), *BRCA2* (*p* = 0.6458), or *PIK3CA* variants (*p* > 0.9999). Of note, seven of eight cases showing CTH events (except IBC19) presented TP53 variants and/or losses.

The comparison of variants in *TP53*, *PIK3CA*, *BRCA2,* MMR, and HR genes with the HRD scores and GII revealed few associations. Variants in HR genes were significantly associated with TAI score (*p* = 0.0318), MMR variants with HRD-LOH (*p* = 0.0117), and *TP53* variants with GII (*p* = 0.0300) (Table S11).

### 2.6. Data Comparison of the Mutational Profile in Non-IBC and IBC Cases

**Figure 4:**
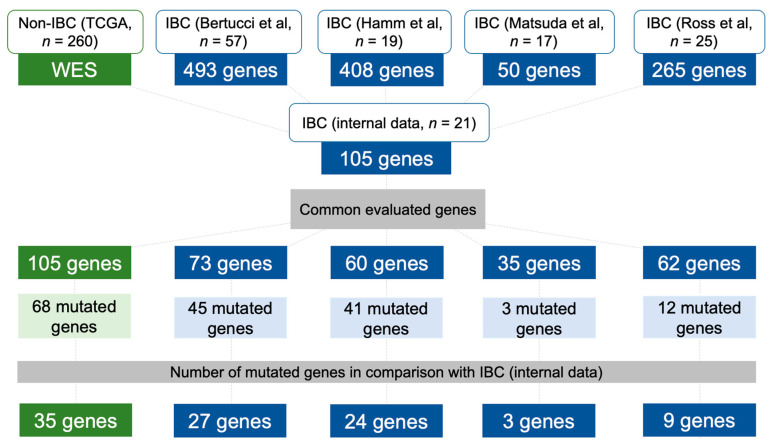
Diagram showing the datasets used to compare the mutational profile of non-IBC and IBC cases with our IBC patients. The total number of genes evaluated by each study, the number of genes in common in relation to our study, the mutated genes per study, and the number of common mutated genes compared to our cases are depicted. WES: whole-exome sequencing.

The mutational profiling of our IBC cases (stages III-IV) was compared with non-IBC stages III-IV from The Cancer Genome Atlas (TCGA, *n* = 260) (Figure 4 and Table S12). Although in different frequencies, 68 of 105 genes presented variants in this comparison. For instance, we found a higher frequency of variants in *EGF, ERBB3*, *HSPH1, MET, RET, MSH3*, *MSH6,* and *PMS2* in our IBC compared with non-IBC stages III-IV from the TCGA (Table S12).

We also compared our variants with four IBC studies that used different targeted gene sequencing panels (Figure 4 and Table S12) [10,18,19,24]. A lower concordance was found between the study of Matsuda et al. [19] and ours: 35 genes in common and three mutated genes. Bertucci et al. [10] evaluated 73 genes in common with ours, while in Ross et al. [18] study, 62 genes were compared (Figure 4 and Table S12).

Two genes, *TP53* and *PIK3CA*, presented a high frequency of variants in all datasets, including the non-IBC from the TCGA cohort (Table S12). A significant prevalence of *BRCA2* variants was observed in 38% of our cases, and in three IBC datasets: 5.3% [10], 20% [18], and 26.3% [24] (Table S12). Interestingly, only 3.1% of TCGA cases presented *BRCA2* variants. We identified only two cases with *NOTCH1* variants (~5% of patients), while Bertucci et al. [10] found 19% of IBC samples with these variants (Table S12).

## 3. Discussion

The molecular mechanisms underlying IBC development and aggressiveness still need to be better explored and understood. We used consolidated molecular approaches to investigate different aspects of IBC biology and its association with clinical parameters. A significant proportion of our cases (~68%) presented a family history of cancer. Twelve women (~35%) had first and/or second-degree relatives with breast or ovarian cancer. The frequency of first-degree relatives with BC among IBC patients was recently estimated as 17% [25]. Although the variant allele frequency and CNA data for tested genes (such as *TP53*, HR, and MMR) indicated that some alterations might be constitutional, germline alterations were not investigated. Recently, Rana et al. [26] assessed the prevalence of germline variants in a cohort of 368 IBC patients. The authors described that *BRCA1*, *BRCA2*, *PALB2,* and *CHEK2* were the most affected genes, mainly detected in TNBC patients (~14%). The presence of germline alterations may represent new therapeutic options for IBC patients.

Most of our cases (~74%) were overweight or obese (BMI > 25 Kg/m^2^). A recent report identified an even more significant proportion of IBC women with overweight or obesity (~80%) [27]. Although our cohort was composed of 11 (~32%) TNBC cases, only five (~15%) were ER-/PR-/HER2+. The prevalence of TNBC and HER2+ IBC patients was reported as being 30% and 40%, respectively [3]. In a cohort of 248 IBC patients, ~17% were classified as hormone receptor-positive/HER2+ [27], while no case of our sample set presented this molecular subtype. Metastasis at diagnosis (13 patients, ~38%) was higher in our cohort than previously reported (30%) [4].

To our knowledge, three studies reported CNAs in IBC using microarrays, and only one used a robust platform (Agilent 244k) [10,16,17]. Gains of 1q, 7q, 8q, 17q, and 20q were frequently detected in our cases. In agreement, gains of 1q, 8q, and 17q were common in IBC [16,17]. Gains covering *MDM4* (1q32.1, ~71%) and *MYC* (8q24.21, 75%) genes were overrepresented in our dataset. In addition to the high frequency of *MDM4* and *MYC* gains, we also identified increased levels of their respective proteins in all 18 IBC samples tested by IHC. Among the samples evaluated by microarray and IHC (eight cases), six presented *MDM4*/*MYC* gains and proteins overexpression. A significant positive correlation was found between *MYC* copy number gains and high scores of C-MYC nuclear distribution. In high-grade BC, a correlation of *MYC* amplification with the percentage of tumor cells with high MYC protein expression was previously observed [28].

In breast cancer, MDM4 inhibits *TP53* transcriptional activity or promotes its degradation through interaction with MDM2 [29,30]. *MDM4* knockdown leads to growth inhibition in different BC subtypes showing *TP53* variants, partially mediated by the downregulation of the CDKN1B (p27) protein [30]. Among the 17 cases with gains covering *MDM4* in our cohort, nine cases also presented *TP53* variants (four of them presented high MDM4 protein expression). Modulation of *MDM4* expression in BC cell lines showed a positive correlation with cell migration and the number of circulating tumor cells compared to orthotopic tumors [31]. It is plausible to suggest that the same mechanisms involved in BC occur in IBC. On the other hand, *MYC* is a well-known oncogene associated with BC progression and a driver candidate in IBC [5,32]. *MYC* amplification was previously reported in 32% of 53 IBC cases evaluated by tNGS [18]. We found *MYC* gains in 18 of 24 IBC cases evaluated by microarray. One case (IBC13) presented *MYC* c.906C > A variant, which could also result in gene activation. A comparison between IBC and non-IBC cell lines showed a specific signature of MYC-driven transcriptional response to TGFβ1 in IBC, while non-IBC were enriched with SMAD3 protein [33]. Recently, it was demonstrated that MYC is a transcriptional target of TP53 in mammary stem cells and is activated in BC as a consequence of *TP53* loss [34]. Based on these findings, *TP53* loss of function (mutation, deletion, or indirectly via *MDM4* amplification) can activate *MYC,* or alternatively, *MYC* is activated by amplification, which contributes to the carcinogenesis and resistance to therapy [32]. A previous study reported that *MYC* amplification with loss of tumor suppressor pathways such as *TP53* and *RB*, can promote poor outcome in TNBC [35]. Similarly, *MYC* overexpression was also correlated with poor prognosis in sporadic BC *BRCA1* mutated [36]. Therefore, the direct or indirect interaction among *TP53, BRCA1, MDM4,* and MYC can increase the tumor aggressiveness, and therapies targeting these pathways are promising strategies for treating IBC. *TERT* gains (5p15.33) have also been associated with more aggressive BC [37]. In our cohort, seven IBC cases had both *TERT* and *MYC* gains. Remarkably, all of them developed distant metastases, and five were classified as TNBC.

We identified frequent losses of 3p, 8p, 9p, 13q, 17p, 19p, and 22q. Common LOH of 3p, 8p, and 13q were also described in IBC samples using microsatellite markers [15]. Two well-known tumor suppressor genes mapped in 13q14.2 and 17p13.1 are involved in the most frequent copy number losses detected in our IBC cases, *RB1* (~58%), and *TP53* (~71%), respectively. TP53 and RB pathways inactivation were associated with resistance to therapy in BC patients, and these genes are drivers in breast carcinogenesis [38]. In a cohort of 53 IBC cases, *TP53* and *RB1* were altered in 62% and 9% of patients, respectively [18]. The authors found only heterozygous deletions of *RB* and homozygous deletions of *TP53* in less than 5% of patients [18]. In agreement, no homozygous deletions of *RB1* and *TP53* were identified in our sample set. However, eight IBC cases from our cohort presented both deletion and *TP53* variants, while one case (IBC15) had both deletion and a *RB1* variant, suggesting that bi-allelic inactivation of these genes also occurs in IBC samples.

Well-known BC-related genes, such as *BRCA1* (IBC11, IBC16, and IBC21), *BRCA2* (IBC7 and IBC11), *PIK3CA* (IBC22 and IBC23), and *TP53* (IBC11, IBC12, IBC16, and IBC22) were covered by cnLOH in our samples. Remarkably, all cases with cnLOH covering *BRCA2* and *TP53* also carried variants in these genes. In *BRCA1* and *BRCA2*-associated hereditary breast and ovarian cancer, cnLOH was reported as the most common LOH mechanism for second hit inactivation [39]. Moreover, cnLOH was more prominent in TNBC than in other molecular subtypes [40]. In agreement, three (IBC11, IBC15, and IBC16) of four IBC cases with more than 10 cnLOH were TNBC.

Among the most prevalent cnLOH found in our cases (3p, 6q, and Xq), we highlight the one in 3p26.3, which includes *CHL1,* a candidate driver gene in IBC. Seven cases presented cnLOH (four TNBC), and six (one TNBC) had losses covering *CHL1*, a putative tumor suppressor gene [41]. *CHL1* hypermethylation and downregulation were associated with increased proliferation and invasion in BC cells, and poor prognosis in BC [41,42]. Interestingly, 11 of 13 IBC patients from our sample set with cnLOH or deletions of *CHL1* developed distant metastases, and 10 of them died from the disease. These data reinforce the involvement of *CLH1* in IBC.

Several inflammatory pathways are described as altered in IBC, such as *NF-κB*, *TGFβ*, *JAK*-*STAT,* and *IL6* pathways [2,43]. Functional enrichment analysis revealed several inflammation-related genes, such as *IL6R*, *IL7*, *CD46,* and *CD55*. IL6R and IL7 may represent potential therapeutic targets in IBC [44,45]. Antibody blockade of IL6R inhibited tumor growth and metastasis in a TNBC mouse xenograft model, while IL7 blockade could prevent the accumulation of immune cells capable of promoting tumor growth [44,45]. Gains covering *IL7* (8q21.23) were more prevalent in our metastatic cases (13/17) than those without metastasis (2/7), suggesting its involvement in the metastatic process. Sixteen cases presented *CD46* and *CD55* gains or amplifications (both mapped on 1q32.3). High expression levels of CD46 and CD55 proteins have been related to poor prognosis in BC [46,47]. Upregulation of *CD46* and *CD55* protects cancer cells from complement-dependent cytotoxicity, and the inhibition of these targets may be used as a strategy to enhance the effect of anticancer antibodies, like trastuzumab in HER2+ BC [48].

A massive genomic rearrangement acquired in a single catastrophic event named chromothripsis was detected in approximately 38% of our IBC samples. We detected CTH affecting chromosomes 8, 11, 13, and 17, as previously described in IBC [10] and BC samples [49,50]. Loss of *TP53* and *MDM4* amplification have been associated with CTH development [51,52]. In ~19,000 samples belonging to 132 cancer types, *TP53* losses were identified in almost 50% of 918 cases having CTH [52]. In our sample set, six of nine CTH samples (~67%) also had *TP53* losses, and all cases with CTH presented *MDM4* gains. CTH events were not enriched in our TNBC cases (2/7, ~29%), which is in agreement with a previous study that reported 25% of CTH in TNBC [49]. CTH has also been related to aggressiveness and poor prognosis [52], but no difference was detected among our patients (metastasis and OS).

Using the genomic alterations detected in our study, we calculated scores/indexes for CIN and HRD. The median GII value was 0.363, which was lower than previously reported (0.435) in almost 900 BC cases from the TCGA database [21]. High GII values were observed in half of our patients. Although GII has been proposed as a predictive marker for clinical outcome [53], no association was found among high GII and TNBC, presence of metastases, or OS in our sample set. A trend toward significance was found between high GII and CTH, an association already reported [49,52]. We evaluated HRD using three independent DNA-based scores (TAI, LST, and HRD-LOH) and the HRD-sum score. The median values of TAI (19.5) and LST (14.5) were higher than previously reported in invasive BC (median TAI and LST values of 12 and 8, respectively) [21]. In contrast, the HRD-LOH median value (2.5) was lower compared to the same study (HRD-LOH of 8) [21]. While TAI and LST scores suggest a higher level of DNA damage repair deficiency in IBC compared with non-IBC, HRD-LOH scores do not seem to quantify HRD in our cases.

Homologous recombination status in BC was better distinguished using a combined score of TAI, LST, and HDR-LOH values [23]. Our combined score (median value of HRD-sum = 41) revealed 11 patients suggestive of having HRD. The highest correlation coefficients were obtained in the comparison of HRD-sum with both TAI (*r* = 0.8536) and LST (*r* = 0.8994). Among the independent scores, a better correlation was obtained between TAI and LST (*r* = 0.7024). In a previous report, TAI and LST also showed a high correlation in eight of 15 cancer types [21]. A significant correlation was also obtained in the comparison of GII with LST, HRD-LOH, and HRD-sum, indicating that HRD contributes to chromosomal instability, as previously reported in sporadic BC [22].

Although not statistically significant, higher median values for TAI, LST, and HRD-sum scores were detected in TNBC patients compared to ER+/PR+ or HER2+ tumors, and in metastatic cases compared to patients without metastases. A previous study with 855 invasive BC from the TCGA database also found higher median values for TAI, LST, and HRD-LOH in TNBC samples than in ER/PR+ and HER2+ subtypes [21]. More recently, Bertucci et al. [10] found significantly higher HRD scores in IBC cases than in non-IBC patients. These findings suggest that aggressive IBC can have higher levels of HRD, and the patients should benefit from platinum and/or PARP inhibitor therapies. We suggest that HRD-based genomic scars may be used in IBC to estimate deficiency in homologous recombination pathways, directing a better therapeutic strategy. We also identified that high scores of TAI were related to a worse prognosis, therefore, HRD score could also be useful as a prognostic marker in IBC.

In summary, the mutational profile of 105 cancer-related genes in 21 IBC patients revealed a high frequency of *TP53* (~52%), *BRCA2* (~43%), and *PIK3CA* (~24%) variants. These three genes were consistently altered in high rates in previous IBC datasets [9,10,18,19,24]. Variants in *BRCA2* were found in much higher percentage of patients (≥20%) in three different datasets, including ours [18,24], when compared with the TCGA non-IBC cases (3.1%), thus suggesting HR deregulation in IBC. Albeit *BRCA1*/*2* deficiency has been related to high HRD-based genomic scars [23,54,55,56], we only observed a trend towards a statistical significance of high TAI and LST scores with *BRCA2* variants. We also found an association between a high TAI score and HR gene variants, which in turn were enriched in metastatic patients and indicates worse OS. A significant proportion of MMR variants was found in our cases (8/21). A recent study reported a similar percentage (43%) of MMR variants in 19 IBC cases [24]. MMR variants have been positively correlated with tumors having higher immune infiltration, which could benefit from immunotherapies, such as PD-L1/PD-1 blockade therapies, an alternative for IBC patient’s treatment [24]. As reported in BC, we also found a link between *TP53* variants and genomic instability (high GII values) [53]. TP53 disruption has been reported as an important mechanism allowing the occurrence of genomic instability, both in vitro and in mouse models. TP53 inactivation may lead to the emergence of complex karyotypes with both numerical and structural aberrations [57]. No association was found between *PIK3CA* variants and the main clinical variables, in contrast to a previous study in IBC that detected an association between worse metastasis-free survival and *PIK3CA* variants [9]. The main limitation of our study is the relatively small number of samples investigated. Nevertheless, IBC is a very rare cancer and our results were deeply explored and compared with external datasets, which represents an additional step to better understand the molecular mechanisms behind IBC emergence and progression.

## 4. Materials and Methods

### 4.1. Patients

Thirty-four unrelated IBC patients were chosen from a retrospective cohort of 136 previously admitted patients at Barretos Cancer Hospital and A.C. Camargo Cancer Center, São Paulo, Brazil, between 2002 and 2014. The Human Research Ethics Committee of both institutions (A.C. Camargo Cancer Center Protocol #2121/15 and Barretos Cancer Hospital Protocol #1145/2016) approved the study. All patients were informed of the procedures and provided written informed consent. The tissue biopsies were collected from primary IBC prior to surgery and radio-chemotherapy treatment. The clinical and histopathological information were retrieved from the medical records updated in January 2020. The staging system was based on the 8th Edition of AJCC (American Joint Committee on Cancer) Cancer Staging Manual recommendations [58], and all IBC cases were classified as T4d. Patients with synchronous or metachronous cancers at the time of diagnosis, having tumors with lymphovascular invasion, and no clinical hallmarks of IBC were excluded. All selected tissues were analyzed by two expert pathologists (C.S.N. and C.A.B.T.O.) and macrodissected using sterile scalpels to scrape off regions of tumors labeled on hematoxylin and eosin-stained tissue sections. Immediately after macrodissection, the biological materials were stored at −80 °C until DNA extraction.

The immunohistochemical (IHC) status of ER (estrogen receptor), PR (progesterone receptor), and HER2 were evaluated in all IBC tissues, as routine diagnostic procedures. ER and PR positivity were defined as nuclear staining in 1% or more of cells [59]. ER and/or PR positive tumors were considered as hormone receptor-positive. For HER2, a complete strong membrane staining intensity (+3) in >10% of cells identified positive samples [60]. Twenty-seven cases presented IHC scores of 0 or +1 and were classified as HER2-negative. Five tumors had an IHC score of +3 and were considered HER2-positive. Only two cases (IBC7 and IBC18) presented the IHC score of 2+ and were investigated by FISH (fluorescence in situ hybridization), which confirmed these cases as HER2-negative.

Immunohistochemistry for MYC and MDM4 proteins were performed in 18 IBC tissues, eight of them also evaluated by CNAs (IBC9, IBC10, IBC16, IBC18, IBC19, IBC20, IBC24, and IBC25) and mutational profile (except IBC19). The DNA was not isolated in the remaining 10 cases due to the lack of fresh-frozen tissues to carry out the genomic experiments.

### 4.2. High-Resolution Chromosomal Microarray

Genomic DNA was isolated (QIAsymphony Kit, Qiagen, Valencia, Ca, USA) from 24 fresh-frozen IBC tissue biopsies containing at least 80% of tumor cells. CytoScan HD Array (Affymetrix, Santa Clara, CA, USA) platform was used to evaluate CNAs and copy-neutral loss of heterozygosity (cnLOH), as previously described [61]. CNAs detected in more than 50% of cases and cnLOH in ≥ 25% of IBC were identified using the CoNVaQ web tool [62]. A functional enrichment analysis, using Gene Ontology (GO) biological process terms was carried out in the WEB-based Gene SeT AnaLysis ToolKit (WebGestalt) 2019 online software considering the genes found in the most common CNAs [63]. The microarray data have been deposited at the Gene Expression Omnibus (GEO, with the accession number GSE144015.

Chromothripsis (CTH) events were detected with the CTLPScanner [64]. We used the following thresholds described by Fontana et al. [65] to consider the CTH event: log10 of likelihood ratio ≥ 8, more than 10 breakpoints, a minimum segment size of 10 kb, and 0.1 as the signal distance between adjacent segments. Events with prevalent CNAs involving ≤10% of the detected region were excluded.

Genomic instability index (GII) (high: >median value) was calculated according to Liu et al. [22]. Three homologous recombination deficiency (HRD) scores measures (TAI, LST, and HRD-LOH) were estimated, as previously described [21]. TAI refers to the number of subtelomeric regions with an allelic imbalance that start beyond the centromere and extend to the telomere region (high: >median value) [54]. LST is based on the number of chromosomal breaks between adjacent regions of at least 10Mb (high: >15 in diploid and >20 in polyploidy tumors) [55]. The number of cnLOH regions larger than 15Mb and shorter than the whole chromosome (high: ≥10) was named HRD-LOH score [56]. We also used a combined HRD score (HRD-sum) obtained by the unweighted numeric sum of TAI, LST, and HDR-LOH scores (high: ≥42), as previously described [66].

### 4.3. Immunohistochemistry (IHC)

Representative areas of 18 IBC biopsies were arrayed in duplicate in a tissue microarray (TMA). Tissue cores of 1 mm^2^ were punched from each IBC biopsy and assembled using the Tissue Microarrayer (Beencher Instruments, Silver Spring, USA). A section of 3µm of paraffin-embedded IBC samples was mounted in glass slides (Fisherbrand-Color Frost^TM^, Fisher Scientific, Pittsburgh, PA, USA). Both the first and last slides were stained with hematoxylin-eosin to confirm the diagnosis. The IHC protocol was performed as previously described [28]. Briefly, the slide sections were dewaxed in xylol and rehydrated in graded ethanol. The mouse monoclonal anti-C-MYC (Clone 9E10, 1:800 dilution; Santa Cruz Biotechnology, Dallas, TX, USA) and rabbit polyclonal anti-MDM4 (Catalog number: polyc04-1555, 1:50 dilution; Merck KGaA, Darmstadt, Germany) were used as primary antibodies. A polymer system (Envision, Dako, Carpinteria, CA, USA) was used as a secondary antibody conjugated to peroxidase and the chromogen 3′-diaminobenzidine tetrahydrochloride (DAB, Dako, Carpinteria, CA, USA), followed by Harris hematoxylin counterstain. C-MYC and MDM4 scores using negative and positive controls were based on Blancato et al. [28] with modifications. Briefly, nuclear and cytoplasmic proteins expression were evaluated separately, with the following scores of distribution (percentage): score 0: no expression, score 1: 1 up to 25% of positive cells, score 2: 26–50%, score 3: 51–75%, and score 4: 76–100%. The intensity of nuclear and cytoplasmic staining was evaluated as mild (score 1), moderated (score 2), and intense (score 3). High expression was defined in cases showing intensity score ≥2 or score of percentage ≥3, while the intensity score of 1 or the percentage score ≤2 was considered as having low expression. Although Blancato et al. [28] defined scores for C-MYC expression, we used a similar approach for MDM4 expression, since both C-MYC and MDM4 are nuclear transcription factors.

### 4.4. Target Enrichment-Next Generation Sequencing (tNGS) and Tumor Burden Assessment

Targeted NGS was carried out in 105 cancer-related genes (Table S9) considering all exons, 3′ UTR and 5′UTR (SureSelect^XT^ Custom Panel, Agilent Technologies, Inc., Santa Clara, CA, USA). Among them, 13 DNA repair genes are involved in the homologous recombination (*ATM*, *BARD1*, *BRCA1*, *BRCA2*, *BRIP1*, *MUS81*, *PALB2*, *POLD1*, *RAD50*, *RAD51B*, *RAD51C*, *RAD51D,* and *XRCC2*) and six in the mismatch repair (MMR) (*MLH1*, *MLH3*, *MSH2*, *MSH3, MSH6,* and *PMS2*) pathways. The libraries were prepared using SureSelect^QXT^ Library Prep Kit (Agilent) and sequenced on the NextSeq 550 system (Illumina, San Diego, CA, USA) according to the manufacturer instructions. Analysis of amplified indexed library DNA was performed using High Sensitivity D1000 ScreenTape (on Agilent TapeStation). Three (IBC3, IBC19, and IBC23) of 24 cases evaluated by microarray were excluded due to DNA quantity and quality, and 21 were multiplexed into 1.4pM pool and loaded onto the NextSeq 550 system (Illumina).

For analysis, raw sequencing reads were demultiplexed, and FASTQ files were obtained. The resulting reads were mapped to the UCSC human genome reference build 19 using the BWA alignment algorithm [67]. Variant calling and quality filtering were performed with Genome-Analysis-Toolkit (GATK) [68]. Variant annotation was primarily performed using ANNOVAR, GATK, and SnpEff [69,70]. The pipeline for variant filtering is depicted in Figure S1B. The variant selection was based on: (1) the allele frequency (<0.01) (2) absent in gnomAD (The Genome Aggregation Database) or ABraOM (Online Archive of Brazilian Mutations) [71,72] (3) variants classified as pathogenic or likely pathogenic in ACMG (American College of Medical Genetics and Genomics), ClinVar or Clinvitae [73,74,75] (4) variants of uncertain significance (VUS) in ACMG presenting loss of function (e.g., frameshift, stopgain, canonical splicesite). Variants classified as synonymous; located in 3′ or 5′ UTR, intergenic or intronic regions or mapped in the homopolymer regions were excluded. The selected variants were manually curated using the Genome Browse software (Golden Helix, Inc., Bozeman, MT, USA). The tNGS data have been deposited at the Sequence Read Archive (SRA, with the accession number PRJNA601891).

### 4.5. Mutational Profile of Non-IBC and IBC Using Independent Validation Datasets

We compared the mutational profile of our internal dataset with non-IBC (TCGA) and IBC cases [10,18,19,24]. We used the cBioPortal for data visualization and genomic analysis of invasive breast carcinomas from TCGA Pan-Cancer data (*n* = 1084 patients) [76,77]. Next, we selected non-IBC stage III and IV from TCGA with mutational data available (*n* = 260). The gene list with variants and the percentage of samples with one or more variants were retrieved using cBioPortal web service [78]. Similarly, we retrieved the mutational data of primary IBC from Bertucci et al. [10] (*n* = 57), Ross et al. [18] (*n* = 25), Matsuda et al. [19] (*n* = 17), and Hamm et al. [24] (*n* = 19) available in the supplementary files. Table S12 shows the frequency of variants in common among our dataset, TCGA, and IBC studies.

### 4.6. Statistical Analyses

The statistical model (Fisher’s exact test) of CoNVaQ was used to test for association between genomic alterations (gains, losses, and cnLOH) and pathological and clinical features [62]. Additional analyses were performed using GraphPad Prism 8.1.0 (GraphPad Software Inc., La Jolla, CA, USA). Fisher’s exact or Chi-square tests were used to compare clinicopathological characteristics, CTH, HRD scores, GII, and gene variants. Spearman correlation was applied to investigate associations among the HRD scores (TAI, LST, HRD-LOH, and HRD-sum) and GII; and also to assess the relationship between *MYC* and *MDM4* copy number states and the C-MYC and MDM4 IHC scores (nuclear/cytoplasmic distribution and intensity) in the eight cases that were evaluated by both microarray and IHC. The coefficient of multiple correlation (correlation matrix and multiple linear regression) was performed to investigate the independent correlation among metastasis, molecular subtypes, HR genes status, and OS. The survival curves were generated using the Kaplan–Meier method, and the statistical significance was determined using the log-rank test. OS was defined as the period (in months) between the date of biopsy and death. Significance was considered with *p* ≤ 0.05.

## 5. Conclusions

We comprehensively explored the molecular and clinical findings of IBC, pointing out genetic alterations that could be used as prognostic or predictive biomarkers in IBC. Frequent gains of *MDM4* and *MYC*, which were translated into increased protein expression levels, are potentially associated with IBC aggressiveness. Moreover, *MDM4* may represent an interesting therapeutic target for IBC treatment. We also found alterations associated with worse prognosis, such as metastasis (including *CHL1* loss, high HRD scores, and HR variants) and shorter overall survival. Particularly, TAI scores might be useful to identify more aggressive IBC, predict deficiency in HR genes, and select patients for platinum or PARP inhibitor therapy. A subset of our patients showing MMR variants might benefit from immunotherapy. Our data have important implications for our understanding of IBC.

## Figures and Tables

**Table 1:** Clinical and pathological characteristics of 34 female patients with inflammatory breast carcinomas evaluated in this study.

Features	Number of Patients (%) ^2^
Age (years)	
<50	10 (29)
≥50	24 (71)
Family history of cancer	
No	9 (26)
Yes	23 (68)
Breast and/or ovarian cancer	12 (35)
Unknown	2 (6)
Body Mass Index (Kg/m^2^)	
≤24.9 (Normal)	7 (20)
25 to 30 (Overweight)	5 (15)
>30 (Obese)	20 (59)
Unknown	2 (6)
Histological grade ^1^	
I	1 (3)
II	14 (41)
III	19 (56)
Clinical stage	
III	21 (62)
IV	13 (38)
Hormone receptor (ER or PR) /HER2 status	
+/+	0
+/−	18 (53)
−/+	5 (15)
TNBC	11 (32)
Distant metastases	
No	8 (24)
Yes	26 (76)
At diagnosis (Stage IV)	13 (38)
During follow-up	13 (38)
Survival status	
Alive	7 (20)
Dead	25 (74)
Loss of follow-up	2 (6)

+, positive; –, negative; ER, estrogen receptor; PR, progesterone receptor; TNBC, triple-negative breast cancer. ^1^ according to Scarf Bloom Richardson grading system. ^2^ DNA was obtained from 24 of 34 patients. Microarray and tNGS were performed in 24 and 21 patients, respectively. Eighteen cases were submitted to immunohistochemistry analysis, eight of them were also evaluated by microarray and targeted next-generation sequencing (except one). Ten cases were used exclusively in the IHC experiments.

**Table 2:** Genetic variants found in *TP53*, *PIK3CA,* homologous recombination (HR), and mismatch repair (MMR) genes detected in 21 inflammatory breast carcinomas assessed by tNGS *.

Cases	*TP53*	*PIK3CA*	HR gGenes	MMR Genes
IBC1		c.3140A>G;p.His1047Arg		
IBC2		c.1633G>A; p.Glu545Lys		
IBC4			*ATM* (c.3887C > G; p.Pro1296Arg) PG, Loss	
IBC5			*BRCA2* (c.4963_4964insA; p.Tyr1655Ter) Loss	
IBC6				
IBC7			*BRCA2* (C.2806_2809delAAAC; p.Ala938Profs) cnLOH	*MLH1* (c.2146G > A; p.Val716Met) PG, Loss
IBC8	c.512A > G; p.Glu171Gly PG, Loss			
IBC9			*BRCA2* (c.5682C > G; p.Tyr1894Ter) Loss *RAD51B* (c.728A > G; p.Lys243Arg) cnLOH *POLD1* (c.1055G > A; p.Arg352His) Gain	
IBC10			*BRCA2* (c.2T > G; p.Met1?)	
IBC11	c.309C > A; p.Tyr103Ter Gain and cnLOH		*BRCA2* (c.316+1G >T) Gain and cnLOH *RAD51B* (c.315+8A > G) PG, Gain and cnLOH	*MSH6* (c.3961A > G; p.Arg1321Gly) PG
IBC12	c.659A > G; p.Tyr220Cys PG, cnLOH		*RAD51D* (c.899G > A; p.Arg300Gln) PG, Loss	*PMS2* (c.2186_2187delTC; p.Leu729Glnfs) PG, Gain
IBC13	c.712T > G; p.Cys238Gly PG		*BRCA2* (c.6988A > G; p.Ile2330Val) PG	
IBC14	c.586C > T; p.Arg196Ter PG, Loss		*BRCA2* (c.8869C > T; p.Gln2957Ter) Loss	
IBC15			*PALB2* (c.43G > T; p.Glu15Ter) cnLOH	*MSH3* (c.1567G > A; p.Glu523Lys) PG *MSH3* (c.1571A > C; p.Asn524Thr) PG
IBC16	c.856G > A; p.Glu286Lys Loss and cnLOH			*MSH3* (c.2436-5C > G Gain
IBC18	c.626_627delGA; p.Arg209Lysfs PG, Loss	c.3140A > T; p.His1047Leu	*BRCA2* (c.9227G > T; p.Gly3076Val) Loss	
IBC20	c.578A > T; p.His193Leu PG c.560-2A > C PG, Loss	c.1035T > A; p.Asn345Lys	*BRCA1* (c.3858delT; p.Ser1286Argfs) PG, Loss	*MLH3* (c.2638C > G; p.Leu880Val) PG
IBC22	c.731G > A; p.Gly244Asp Loss and cnLOH	c.3140A > G; p.His1047Arg	*MUS81* (c.416G > A; p.Arg139Gln) PG	*PMS2* (c.2383G > A; p.Asp795Asn) PG
IBC24	c.844C > T; p.Arg282Trp PG, Loss			*MSH6* (c.1406A > G; p.Tyr469Cys) PG
IBC25				
IBC26	c.542G > A; p.Arg181His PG, Loss:			

* Copy number alterations and Variant Allele Frequency (VAF) of *TP53*, HR and MMR genes presenting genetic variants are shown. (c.) and (p.) indicate the coding sequence and the amino acid position according to HGVS (Human Genome Variation Society), respectively. cnLOH, copy number loss of heterozygosity; PG, potentially germline variant (VAF, <0.5). Copy number loss < 2.0; Copy number gain > 2.0.

## Supplementary Materials

The following are available online at https://www.mdpi.com/2072-6694/12/10/2816/s1, Figure S1: (A) Pipeline for variant selection and classification. (B) Complete basic and annotation pipelines for targeted next-generation sequencing (tNGS) analysis, Table S1: Complete description of all copy number alterations and cnLOH identified in 24 inflammatory breast carcinomas enrolled in this study, Table S2: Genomic gains and losses identified in at least 12 inflammatory breast carcinomas, and cnLOH detected in at least six tumor samples, Table S3: Top 10 non-redundant biological process terms identified in genes covered by the most common regions involved in copy number alterations, Table S4: Gains frequently detected in TNBC (*n* = 7) compared to hormone receptor+ and/or HER2+ IBC cases (*n* = 17), Table S5: Genomic gains frequently identified in metastatic (*n* = 17) compared to non-metastatic cases (*n* = 7), Table S6: Chromothripsis-like pattern identified in nine of 24 inflammatory breast cancer cases, Table S7: Genomic instability index and homologous recombination deficiency score values for each tumor sample evaluated in this study, Table S8: Spearman correlation test for the HRD-associated scores and GII, Table S9: List of 105 genes evaluated by targeted next-generation sequencing (tNGS) in inflammatory breast carcinomas, Table S10: List of variants identified in 21 inflammatory breast carcinomas analyzed by targeted next-generation sequencing (tNGS), Table S11: Association analyses of specific mutations with high scores of HRD measures and GII, Table S12: List of genes affected by variants and percentage of altered cases comparing our internal dataset (21 IBC) with non-IBC cases from TCGA, and IBC patients from four different studies.
