# Supplementary figures and images for: Inflammatory Breast Cancer: Clinical Implications of Genomic Alterations and Mutational Profiling
Source: Cancers (Basel). 2020 Sep 30;12(10):2816. doi: 10.3390/cancers12102816 (PMC7650681; doi:10.3390/cancers12102816)

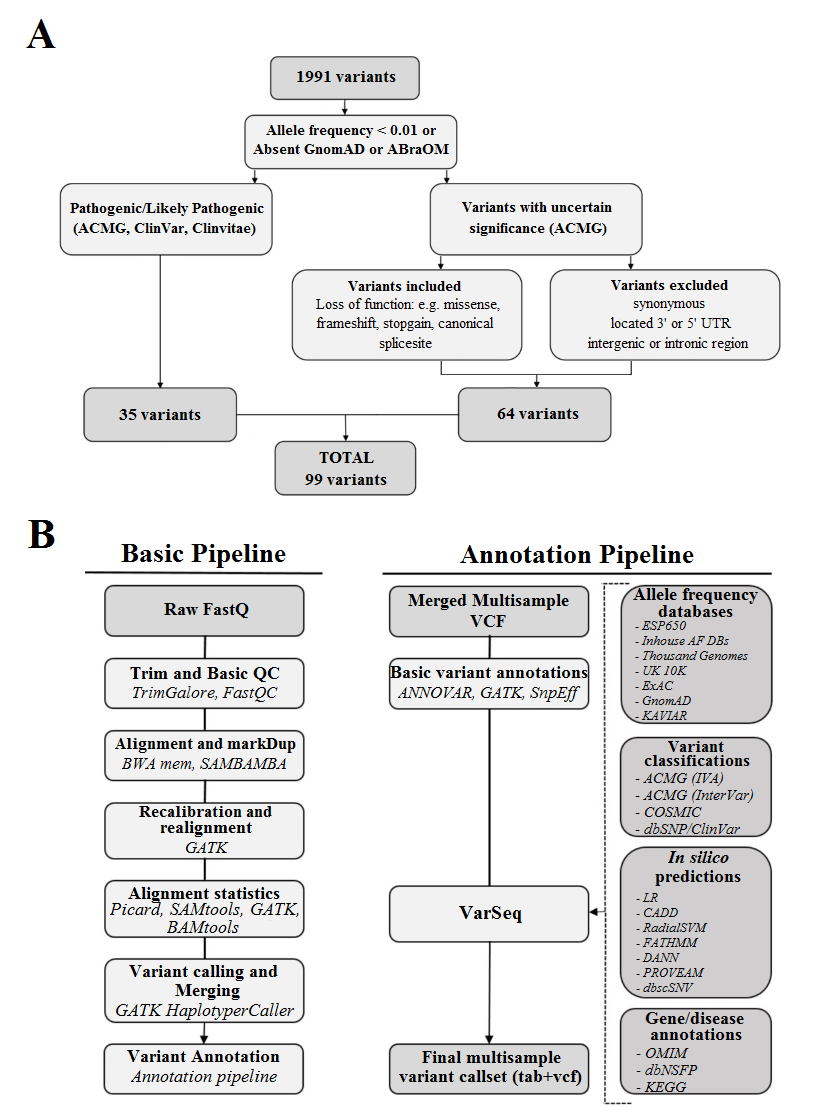

Supplement: Supplementary file 1 [file cancers-12-02816-s001.zip › Figure S1.tif]
